# Biologics for bone regeneration: advances in cell, protein, gene, and mRNA therapies

**DOI:** 10.1038/s41413-025-00487-0

**Published:** 2026-01-12

**Authors:** Claudia Del Toro Runzer, Elizabeth R. Balmayor, Martijn van Griensven

**Affiliations:** 1 Department of Cell Biology-Inspired Tissue Engineering, MERLN Institute for Technology-Inspired Regenerative Medicine, Maastricht, the Netherlands; 2 Experimental Orthopaedics and Trauma Surgery, Department of Orthopaedic, Trauma, and Reconstructive Surgery, RWTH Aachen University Hospital, Aachen, Germany; 3 Musculoskeletal Gene Therapy Laboratory, Rehabilitation Medicine Research Center, Mayo Clinic, Rochester, MN USA

**Keywords:** Bone, Pathogenesis

## Abstract

Bone fractures represent a significant global healthcare burden. Although fractures typically heal on their own, some fail to regenerate properly, leading to nonunion, a condition that causes prolonged disability, morbidity, and mortality. The challenge of treating nonunion fractures is further complicated in patients with underlying bone disorders where systemic and local factors impair bone healing. Traditional treatment approaches, including autografts, allografts, xenografts, and synthetic biomaterials, face limitations such as donor site pain, immune rejection, and insufficient mechanical strength, underscoring the need for alternative strategies. Biologic therapies have emerged as promising tools to enhance bone regeneration by leveraging the body’s natural healing processes. This review explores the critical role of conventional and emerging biologics in fracture healing. We categorize biologic therapies into protein-based treatments, gene and transcript therapies, small molecules, peptides, and cell-based therapies, highlighting their mechanisms of action, advantages, and clinical relevance. Finally, we examine the potential applications of biologics in treating fractures associated with bone disorders such as osteoporosis, osteogenesis imperfecta, rickets, osteomalacia, Paget’s disease, and bone tumors. By integrating biologic therapies with existing biomaterial-based strategies, these innovative approaches have the potential to transform clinical management and improve outcomes for patients with difficult-to-heal fractures.

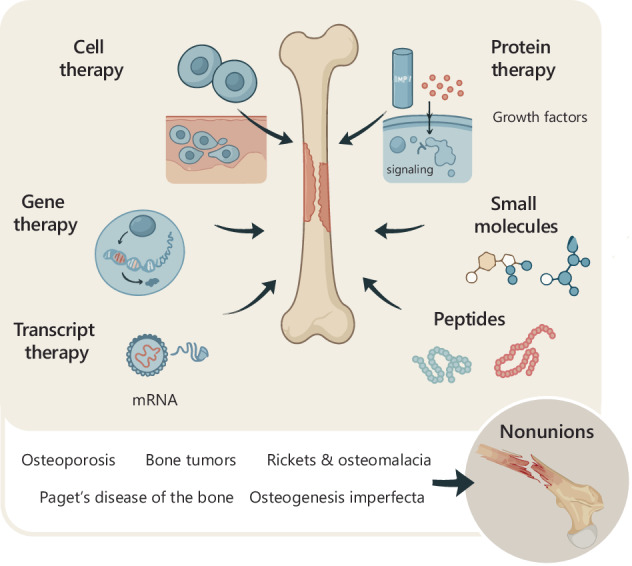

## Introduction

Bone fractures are a prevalent medical condition, with an estimated 178 million new fractures occurring globally each year.^1^ Despite advances in medical treatment, 5%–10% of fractures result in nonunion, a condition where the bone fails to heal properly.^2^ The FDA defines a nonunion as a fracture that shows no visible signs of healing at least nine months post-trauma.^3^ This condition can lead to prolonged disability and significantly impact patients’ quality of life.

The risk of nonunion formation is influenced by factors such as fracture size, mechanical instability, shear stress, poor vascularization, and infections.^4^ In patients with bone disorders, this risk is further increased due to a diminished pool of osteogenic precursors with impaired proliferative capacity, altered bone mineral matrix composition, and local or systemic disruptions in hormonal and growth factor signaling.^5–8^

Managing nonunion fractures remains challenging and costly, with treatment expenses ranging from €8 000 to €90 000 per patient until complete healing is achieved.^9,10^ The gold standard remains autologous bone grafting, which uses the patient’s own bone—typically harvested from the iliac crest—due to its high osteogenic potential and lack of immune rejection.^11^ However, this procedure is limited by donor site morbidity, including chronic pain, infection, and nerve or muscle injury, as well as constraints in graft availability and shape.^12,13^

To address bone deficits, allografts and xenografts have been explored as alternatives. Allografts, sourced from living donors or cadavers, offer immediate availability in various sizes and shapes,^14,15^ but have reduced osteogenic potential compared to autografts.^16^ Similarly, xenografts, typically derived from bovine, porcine, or coral sources, offer osteoconductive properties at a lower cost. However, bovine and porcine xenografts carry risks of immune rejection and disease transmission,^17,18^ while coral-derived xenografts are limited by poor resorption rates.^19^

Synthetic biomaterials, such as calcium phosphate ceramics and cements, offer further options for bone defect treatment due to their composition, which mimics the native bone matrix and supports osteoconduction.^20,21^ While effective for small defect reconstruction, these materials are often unsuitable for larger defects due to limited mechanical strength and insufficient neovascularization.^4,22^

Biologic therapies have emerged as a promising approach to enhancing bone healing by leveraging biological molecules that support the body’s natural regeneration mechanisms. Among these, growth factors and cell-based therapies have shown encouraging clinical results. However, challenges such as limited efficacy, immune responses, and high production costs necessitate further advancements in the field.

Gene and transcript therapies, which are DNA or RNA based, respectively, represent a novel frontier in regenerative medicine for bone repair.^23^ Gene therapy involves the introduction, alteration, or removal of genes to treat or prevent a disease, while transcript therapy focuses on the delivery of RNA transcripts to modulate gene expression.^24,25^ By directly delivering genetic material or its transcribed mRNA into the fracture site, these therapies enable the localized production of therapeutic proteins, promoting and accelerating osteogenesis and vascularization.^25^ Currently, most transcript-based strategies rely on chemically modified mRNA, owing to its enhanced translational efficiency, reduced immunogenicity, and tunable protein expression, with an improved safety profile and affordable costs.

Recent breakthroughs in gene delivery platforms, including lipid- and polymer-based vectors as well as lipid nanoparticles, have enhanced the stability, bioavailability, and cellular uptake of nucleic acids, overcoming key barriers to clinical translation.^26–28^ Furthermore, biomaterial scaffolds engineered to carry gene or transcript therapies have improved their targeted application, ensuring optimal integration into the bone microenvironment.^29^

This review provides a comprehensive overview of the current landscape of biologic therapies for nonunion fractures. We begin by outlining the key stages of fracture healing, highlighting the main cell types and growth factors involved in the repair process. Next, we categorize biologic therapies that are either under investigation or in clinical use, including protein therapy, gene therapy, transcript therapy, cell therapy, peptides, and small molecules. Each therapeutic category is explored in terms of its distinct mechanism of action, therapeutic advantages, limitations, and clinical relevance.

Furthermore, we discuss the potential applications of these biologic therapies across diverse patient populations, with a focus on individuals affected by osteoporosis, osteogenesis imperfecta, rickets, osteomalacia, bone tumors, and Paget’s disease. By evaluating the efficacy and safety profiles of these therapies in various pathological contexts, we aim to underscore their transformative potential in addressing the unmet needs of bone regeneration and fracture healing.

## The fracture healing process: key cellular and growth factor contributors

**Fig. 1:**
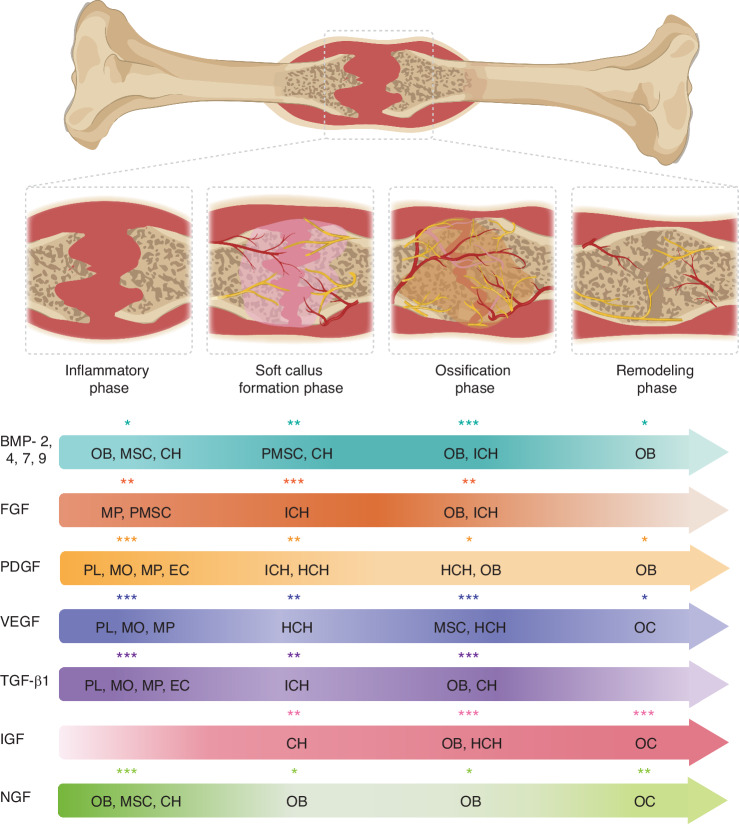
Expression profile of osteogenic growth factors during fracture healing. This schematic illustrates the four phases of bone healing: inflammatory phase, soft callus formation, ossification, and remodeling. At each stage, specific cell populations locally and focally express osteogenic growth factors. The asterisks and intensity of each arrow represent the expression level of a given factor by a particular cell type. OB osteoblasts, MSC mesenchymal stem cells, CH chondrocytes, PMSC periosteal mesenchymal stem cells, ICH immature chondrocytes, MP macrophages, PL platelets, MO monocytes, EC endothelial cells, HCH hypertrophic chondrocytes, OC osteoclasts, BMP bone morphogenetic protein, PDGF platelet-derived growth factor, VEGF vascular endothelial growth factor, FGF fibroblast growth factor, TGF-β1 transforming growth factor-β1, IGF insulin-like growth factors, NGF nerve growth factor

Fracture healing can be divided into four phases, each characterized by the presence of different cells, extracellular matrix (ECM) components and growth factors (Fig. 1). These sequential phases include the inflammatory phase, soft callus formation, ossification, and remodeling.^30^ Physiological growth factors are primarily stored in bone ECM and are actively released upon injury to initiate the regeneration process.^31^ These growth factors exert their effects by binding to transmembrane receptors, triggering intracellular signaling cascades that activate specific transcription factors and gene expression programs.^32^

In the inflammatory phase, vascular disruption leads to hematoma formation containing bone fragments and immune cells.^33^ Peaking within 48 h and subsiding by one week, this phase immobilizes the fracture through pain-induced restriction and swelling.^34^ The acidic microenvironment triggers platelet degranulation, coagulation, and growth factor release. Concurrently, immune cells secrete cytokines that promote osteogenesis, angiogenesis, and initiate tissue repair.^35^

The soft callus formation phase lasts for several weeks. During this time, mesenchymal stem cells (MSCs) differentiate into fibroblasts, chondroblasts, and osteoblasts.^36^ Fibroblasts produce ECM proteins like type I and II collagen, while chondroblasts and osteoblasts form a cartilaginous callus that stabilizes the fracture, providing initial mechanical support.^37,38^ As local pH shifts from acidic to slightly alkaline, the enzyme alkaline phosphatase (ALP) facilitates mineral deposition and bone formation.^39^

During the ossification phase, bone forms through intramembranous and endochondral ossification. Intramembranous ossification primarily occurs in the periosteal region of the fracture, specifically at the fracture periphery.^40^ It involves direct osteoblast-mediated ECM secretion, primarily composed of type I collagen and osteocalcin, and calcium phosphate deposition, leading to osteoblast differentiation to osteocytes.^41^ Endochondral ossification, occurring predominantly in the central regions of the fracture, relies on chondrocyte-produced collagen type II matrices, which are later replaced by bone tissue predominantly containing type I collagen.^42^

Finally, the remodeling phase converts immature, weak woven bone into organized lamellar bone.^43^ In this process, osteoblasts, osteocytes, and osteoclasts work together to restore bone structure and mechanical strength, a process that can last months to years.^44^

Given that these signaling pathways regulate multiple genes simultaneously, individual growth factors can exert pleiotropic effects within and across different cell types.^45^ Biologic therapies leverage these endogenous mechanisms by delivering specific growth factors at the site of injury to stimulate and accelerate healing.^46,47^ Key growth factors for bone regeneration are shown in Table 1, and its specific role in the fracture healing process is explained in the next sections.

**Table 1:** Growth factors in fracture healing

Growth Factor	Role in Bone Healing	Cellular Expression	Clinical Relevance
Bone morphogenetic proteins (BMPs)	- Differentiates MSCs to osteogenic lineage. - Induces maturation of osteoblasts.^48^	MSCs, osteoblasts, and chondrocytes.^49–51,264^	FDA-approved for clinical use: - rhBMP-2 (INFUSE).^86^ - rhBMP-7 (OP-1 Putty), removed from market.^265^
Fibroblast growth factors (FGFs)	- Promotes blood vessel formation.^266^ - Stimulates type 4 collagenase.^30^	Inflammatory cells, osteoblasts, and chondrocytes.^30^	Not yet FDA approved, clinical trials report accelerated healing using rhFGF-2 for: - high tibial closed wedge osteotomies.^88^ - tibial fractures.^267^
Platelet-derived growth factor (PDGF)	- Promotes angiogenesis.^55^ - Enhances recruitment and proliferation of MSCs.^56^	Platelets, monocytes, macrophages, and endothelial cells.^56^	FDA approved rhPDGF-BB for use in hindfoot and ankle fusion.^268^
Transforming growth factor-β1 (TGF-β1)	- Induces MSC recruitment and differentiation.^61^ - Promotes angiogenesis and cartilage formation.^64^ - Modulates cartilage matrix calcification.^269^	Platelets, macrophages, osteoblasts, osteoclasts, and chondrocytes.^52,62^	- Not FDA approved. - In mice, rats and rabbits, promotes osteogenic differentiation of MSCs, but higher concentrations inhibit the process.^270^
Insulin-like growth factors (IGFs)	- Stimulate osteoblast precursor proliferation.^65^	MSCs, osteoblasts, osteoclasts, and chondrocytes.^66^	- FDA approved but not for bone regeneration - Its administration showed increased bone mass in women aged 65-90 years with hip fracture.^271^
Vascular endothelial growth factor (VEGF)	- Restores vascular networks.^71^ - Supports new bone formation.^272,273^	Immune cells, hypertrophic chondrocytes, preosteoblasts, and osteoclast.^71,274^	- FDA approved but not for bone regeneration. - It enhanced bone formation and angiogenesis in diverse preclinical models.^275–277^
Nerve growth factor (NGF)	- Induces nerve growth, differentiation and survival.^73,74^ - Stimulates angiogenesis, and promotes osteogenesis.^79^	MSCs, osteoclasts, osteocytes, chondrocytes, and synovial fibroblasts.^80–82^	In preclinical studies, local administration of NGF promotes osteogenesis, and synthesis of VEGF.^278^

### Bone morphogenetic proteins (BMPs)

BMPs are acknowledged as the most efficient growth factors to support the healing of large bone defects.^48^ During fracture healing, BMP-2, BMP-4, BMP-7, and BMP-9 are strongly expressed in undifferentiated MSCs during the inflammatory phase.^49^ These BMPs are also highly expressed in proliferating osteoblasts during intramembranous ossification^50^ and in proliferating chondrocytes during chondrogenesis and endochondral ossification. Their expression decreases in mature chondrocytes but remains high in osteoblasts near the endochondral ossification front.^51^ Additionally, BMPs regulate the expression of other critical growth factors, including insulin-like growth factors (IGFs) and transforming growth factors (TGFs), thereby orchestrating bone formation. Beyond osteogenesis, BMPs also contribute to angiogenesis, particularly BMP-4, which enhances vascularization through interactions with type I and IV collagen and heparin.^30^

### Fibroblast growth factors (FGFs)

FGFs constitute a large family of signaling proteins that play crucial roles in tissue development, metabolism, and bone repair. Among them, FGF-1 and FGF-2 are the most extensively studied in the context of bone metabolism. During the inflammatory phase of fracture healing, FGF-1 is expressed in macrophages and periosteal cells. As healing progresses, it is detected in osteoblasts during intramembranous ossification and in immature chondrocytes during chondrogenesis.^30^ In endochondral ossification, its expression is limited to osteoblasts. In contrast, FGF-2 shows consistent expression throughout regeneration, found in macrophages, osteoblasts, and chondrocytes, during the ossification phase.^30,52^ FGFs also regulate angiogenesis by upregulating VEGF and enhance bone formation by interacting with BMP-2.^53^ FGF-2 deficiency impairs BMP-2 function, while higher BMP-2 levels enhance FGF-2 osteogenic potential.^54^

### Platelet-derived growth factors (PDGFs)

PDGF is a signaling molecule with receptor tyrosine kinase activity that plays a critical role in angiogenesis and the proliferation of MSCs.^55,56^ Initially, PDGF expression is weak during the inflammatory phase but increases and remains stable throughout the repair process, contributing to both cartilage formation and intramembranous bone development.^52^ PDGF acts as a potent chemoattractant for pericytes, facilitating their detachment from the vessel wall, allowing them to transition into MSCs. These MSCs, in turn, are stimulated by PDGF to differentiate into osteoblastic progenitors, thereby enhancing bone regeneration.^57^

Despite its well-established involvement in the fracture healing cascade, the therapeutic potential of PDGF in bone repair remains controversial. For instance, even when combined with osteoconductive materials, PDGF has not consistently demonstrated significant enhancement of bone regeneration in clinical or preclinical models.^58,59^ Furthermore, emerging evidence suggests that PDGF may antagonize the osteoinductive activity of BMP-2.^60^ These findings underscore the complexity of PDGF’s role in bone healing and highlight the need for a more nuanced understanding of its mechanisms of action.

### Transforming growth factor-β1 (TGF-β1)

TGF-β1 is one of the most abundant cytokines in the bone matrix, existing in an inactive precursor form until it is cleaved and activated by acidic conditions or osteoblast-mediated processing.^50^ Once active, TGF-β1 functions as a chemotactic agent to recruit local MSCs for osteogenic differentiation.^61^ At the fracture site, TGF-β1 is produced by various cell types, including platelets, macrophages, osteoblasts, osteoclasts, and chondrocytes.^52,62^ Extracellularly, it is present in the hematoma immediately after injury, playing an essential role in early bone healing.^63^ During intramembranous ossification, TGF-β1 is highly expressed in proliferating osteoblasts, while during chondrogenesis and endochondral ossification, its expression peaks in proliferating chondrocytes.^50^ Additionally, TGF-β1 enhances the production of key bone and cartilage extracellular matrix components, including various collagen types, fibronectin, osteopontin, osteonectin, thrombospondin, proteoglycans, and ALP.^64^

### Insulin-like growth factors (IGFs)

IGFs, also known as somatomedins or sulfation factors, function as key autocrine and paracrine regulators in bone metabolism. IGFs produced by bone tissue become sequestered within the bone matrix and are later released during bone resorption or fracture, where they stimulate osteoblast precursor proliferation via activation of the mTOR signaling pathway.^65^ Outside the liver, bone is the richest source of IGF-1 and IGF-2 in mammals, where these locally produced IGFs play a key role in skeletal development and remodeling. In particular, IGF-1 is essential for creating the osteogenic microenvironment required for MSCs to differentiate into osteoblasts.^65^ Unlike other growth factors involved in fracture healing, IGF-1 mRNA is not detected during the inflammatory phase. Instead, its expression is observed in osteoblasts and prehypertrophic chondrocytes during intramembranous ossification.^66^ IGF-2 mRNA, is expressed in certain osteoclasts and osteoblasts during the remodeling phase, though most osteoblasts remain negative for IGF-2 expression.^66^ Both IGF-1 and IGF-2 contribute to bone formation by enhancing collagen synthesis and reducing collagen degradation.^67^

### Vascular endothelial growth factor (VEGF)

Fractures often disrupt the local blood supply, necessitating rapid revascularization to deliver oxygen and nutrients. VEGF plays a pivotal role in angiogenesis during fracture healing.^68^ Its expression is initiated during the inflammatory phase, driven by hypoxic conditions and the fragmented bone matrix within the hematoma, which stimulates its release from platelets and infiltrating immune cells.^69,70^ Beyond its angiogenic function, VEGF also regulates inflammatory responses, contributing to a balanced and controlled repair environment.^71^ Although cartilage is inherently avascular, studies have demonstrated that hypertrophic chondrocytes actively express and secrete VEGF, facilitating the transition from cartilage to bone.^38^ VEGF further acts synergistically with BMPs to enhance preosteoblast differentiation and metabolism. Additionally, during the remodeling phase, VEGF promotes osteoclast recruitment, survival, and activity, all of which are essential for bone resorption and structural refinement.^72^

### Nerve growth factor (NGF)

Mature bone is a highly innervated tissue, and accumulating evidence suggests that nerve-derived signals play a crucial role in bone regeneration.^73,74^ Several studies have reported a significant upregulation of NGF immediately after bone injury, highlighting its importance in the fracture healing process.^75,76^ Experimental nerve resection via nonspecific surgical methods has been shown to impair fracture healing in mouse models, further underscoring NGF’s role in bone regeneration.^77,78^ Beyond its well-established function in promoting neuronal growth and extension, NGF enhances osteogenic activity, potentially expediting fracture repair.^79^ It has been shown to stimulate osteoblast mitosis and actively participate in skeletal development and ossification. NGF is expressed in MSCs,^80^ osteoclasts, osteocytes, chondrocytes, and synovial fibroblasts.^81,82^

## Categories of biologic therapies

**Fig. 2:**
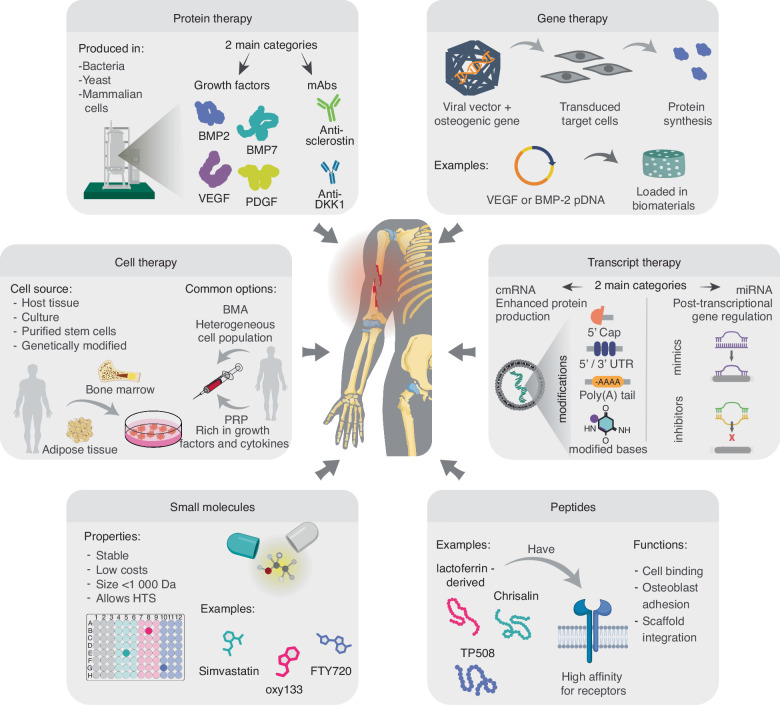
Categories of biologic therapies for bone regeneration. Schematic overview of representative examples across distinct classes of biologic therapies for bone regeneration, including protein therapy, gene therapy, transcript therapy, cell therapy, small molecules, and peptides. Some therapies, such as recombinant BMP-2 and BMP-7, are FDA-approved, while others—like VEGF pDNA gene therapy—are currently in clinical trials. Emerging approaches such as transcript therapy (e.g., cmRNA) remain in the preclinical stage. BMP bone morphogenetic protein, PDGF platelet-derived growth factor, VEGF vascular endothelial growth factor, pDNA plasmid DNA, HA hydroxyapatite, BMA bone marrow aspirate, PRP platelet-rich plasma, cmRNA chemically modified mRNA, HTS high-throughput screening, RGD arginine-glycine-aspartic acid tripeptide

**Fig. 3:**
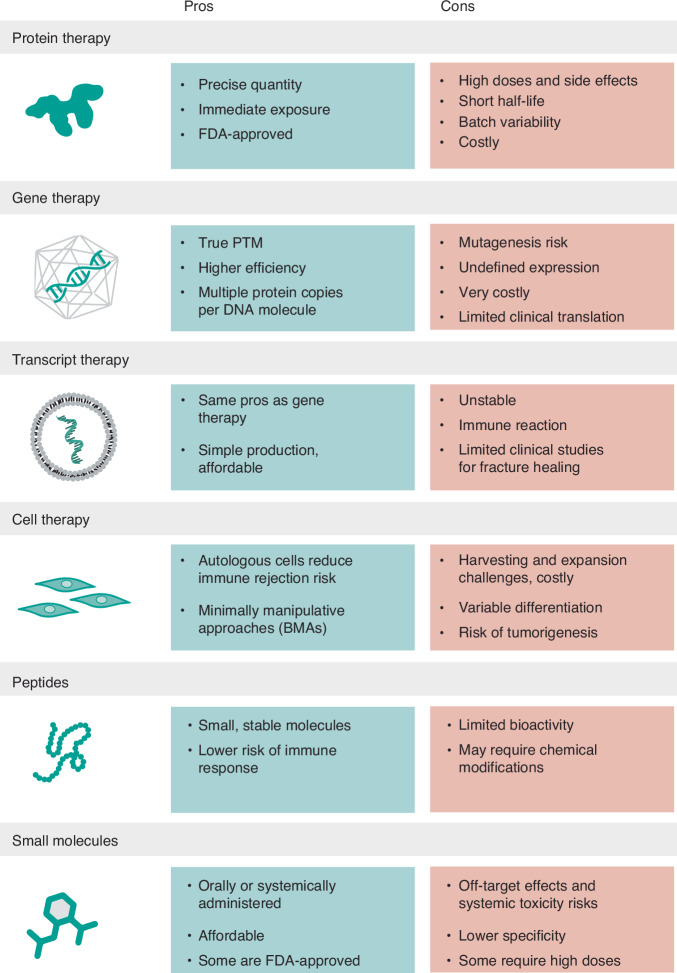
Comparative overview of the advantages and limitations of various biologic strategies for bone regeneration, including protein, gene, transcript, cell, peptide, and small molecule therapies. PTM post-translational modifications, BMAs bone marrow aspirates

Advancements in biologic therapies are continually enhancing the ability to regulate fracture healing pathways, providing innovative treatment options for bone regeneration. According to the FDA, biologics encompass a diverse range of therapeutic products, including recombinant proteins, gene and transcript-based therapies, small molecules, peptides, and cell-based treatments, and blood-derived products (Fig. 2). The following sections will explore each category in the context of bone healing, highlighting their mechanisms, benefits, and challenges (Fig. 3).

### Protein therapy

Recombinant protein therapy involves the direct administration of purified proteins to promote bone regeneration.^83^ These proteins are produced by inserting the gene encoding the desired protein into host cells—such as bacteria, yeast, or mammalian cells—which are then cultured in bioreactors under controlled conditions. The resulting protein is harvested, purified through multiple chromatography steps, and subjected to rigorous quality control assessments to ensure therapeutic efficacy and safety.

Numerous clinical studies have explored the use of recombinant proteins for fracture healing, with BMP-2 and BMP-7 among the most extensively studied and being among the few FDA-approved osteoinductive growth factors for bone regeneration. Commercially available products such as INFUSE® (rhBMP-2, Medtronic) are approved for indications like open tibial fractures and spinal fusions, though they are also used off-label for treating nonunions and in select dental procedures.^84–86^ Other growth factors, such as FGF-2,^87,88^ PDGF,^89–91^ and IGF-1^92,93^ have also been investigated for their roles in stimulating bone repair or improving bone mineral density.^94^

Despite their comparable efficacy to autografts, recombinant protein therapies often require supraphysiological doses to achieve therapeutic effects. In clinical settings, these doses can exceed natural levels by several orders of magnitude, which increases efficacy but also elevates the risk of adverse effects. BMP-2 and BMP-7, for example, have been associated with ectopic bone formation, inflammation, and nerve compression.^84–86^

In addition to growth factors, parathyroid hormone (PTH) and its synthetic analogs, such as teriparatide (PTH(1–34), Forteo®) and abaloparatide (a PTHrP analog), have also been used as anabolic agents in bone regeneration.^95,96^ Intermittent PTH administration transiently activates PTH1 receptors on osteoblasts and osteocytes, triggering intracellular signaling cascades (e.g., cAMP/PKA, PLC/PKC) that promote osteoblast survival, differentiation, and bone-forming activity.^97^ Clinical trials investigating PTH-based strategies for fracture repair and nonunions have produced mixed results. Some studies have reported enhanced healing rates and reduced time to union in specific fracture types (e.g., vertebral, sternal, tibial, and femoral fractures),^98–101^ while others have shown no significant advantage in long bone nonunions compared to placebo or conventional care.^102,103^ In clinical practice, its application in nonunions is often off-label, particularly in patients with metabolic bone disorders or delayed healing, where an anabolic boost is desired.^104^

The Wnt/β-catenin signaling pathway also represents a highly promising target for bone regenerative therapies. Wnt proteins bind to Frizzled receptors and LRP5/6 co-receptors on the surface of osteoprogenitor cells, thereby inhibiting the β-catenin destruction complex. This results in β-catenin stabilization, nuclear translocation, and activation of osteogenic gene expression.^105^ Activation of this pathway promotes osteoblast differentiation and bone formation.^106^ In a study by Minear et al., liposomal Wnt3a was delivered to skeletal defects in mice, stimulating progenitor cell proliferation and accelerating osteogenic differentiation, ultimately enhancing bone regeneration.^107^ Given the central role of this pathway, multiple therapeutic strategies targeting various components of Wnt/β-catenin signaling have been developed—these are further explored in the following section.

### Monoclonal antibodies in bone regeneration

Monoclonal antibodies (mAbs) represent another class of protein-based therapeutics that differ from conventional recombinant proteins, such as BMPs, in their mechanism of action. Instead of replacing or supplementing missing proteins, mAbs function by binding to specific molecular targets involved in bone remodeling, thereby modulating cellular pathways with high specificity. For example, sclerostin, a glycoprotein secreted by osteocytes, is a negative regulator of bone formation by inhibiting the Wnt/β-catenin signaling pathway. Anti-sclerostin antibodies neutralize sclerostin, thereby disinhibiting Wnt signaling and promoting osteoblast proliferation, differentiation, and bone formation, while also reducing bone resorption.

One advantage of monoclonal antibodies is their ability to be administered systemically, allowing them to reach fracture sites through circulation and exert their effects on bone cells throughout the skeleton. For example, Pui Kit Suen et al. investigated the effects of systemic administration (subcutaneous injection, 25 mg/kg, two times per week) of sclerostin antibody (SclAb) on fracture repair in a rat femoral osteotomy model.^108^ SclAb treatment significantly increased bone mineral density (BMD) and enhanced neovascularization. Mechanical testing at weeks 6 and 9 demonstrated significantly higher ultimate load in the SclAb-treated group, indicating improved bone strength.^108^

Clinically, monoclonal antibody therapies have shown promise in difficult-to-heal fractures. A case report by Takuya Uemura et al. described a 61-year-old heavy smoker with a distal radius nonunion who achieved successful bone healing through a combination of distraction plate fixation, graft substitutes, and systemic administration of romosozumab—a monoclonal antibody targeting sclerostin.^109^ The study concluded that combining β-tricalcium phosphate bone grafting with bridge plating and systemic romosozumab can be an effective treatment option for complex nonunion cases, particularly in patients with risk factors such as smoking, diabetes, and bone fragility.^109^

However, the clinical use of romosozumab has raised safety concerns due to an observed increase in serious cardiovascular events, including myocardial infarction and stroke. In clinical trials, patients receiving romosozumab exhibited a higher incidence of these adverse effects compared to those treated with alendronate.^110^ As a result, this therapy should be used with caution in patients with a history of cardiovascular disease or other risk factors. Moreover, the therapeutic potential of romosozumab in fracture healing remains debated. A phase 2 double-blind, randomized clinical trial in patients with acute unilateral tibial diaphyseal fractures found no significant improvement in healing time, regardless of the dosage administered.^111^ Similar results were reported in a second phase 2 dose-finding study involving patients with hip fractures, where romosozumab also failed to accelerate fracture healing.^112^

While anti-sclerostin antibodies remain the most clinically advanced class of anabolic agents for bone, with potential applications in fracture healing and nonunion, other therapeutic targets—such as Dickkopf-1 (DKK1)—are under active investigation. DKK1 is a secreted glycoprotein that inhibits the canonical Wnt/β-catenin signaling pathway by binding to LRP5/6 co-receptors, thereby preventing Wnt ligand binding and suppressing osteoblast development and activity.^113,114^ Anti-DKK1 antibodies counteract this inhibition, allowing Wnt signaling to proceed and stimulating bone formation.^115^ Although still in the preclinical stage, primarily in rodent models, studies have shown that anti-DKK1 antibodies enhance callus formation, improve mechanical strength, and accelerate endochondral ossification in bone fracture models.^116^

Another promising therapeutic is denosumab, a monoclonal antibody that targets RANK Ligand (RANKL)—a key regulator of osteoclast differentiation and activity.^117^ By blocking RANKL-RANK interactions, denosumab effectively inhibits osteoclast-mediated bone resorption.^118^ Although primarily used in the treatment of osteoporosis,^119^ its application in nonunion cases is gaining attention, particularly where excessive or dysregulated bone resorption impairs healing. In a prospective study involving 43 patients with aseptic tibial shaft nonunions following intramedullary nailing, the addition of subcutaneous denosumab (60 mg) postoperatively to plate augmentation and autologous bone grafting significantly reduced the average time to union from 14.82 weeks to 9.45 weeks, with no major complications reported.^120^ While denosumab appears to enhance early callus consolidation, its inhibitory effect on bone resorption may result in delayed remodeling, with woven bone persisting longer before transitioning to mature lamellar bone. Further studies are needed to fully understand the long-term impact of denosumab on bone remodeling and its overall therapeutic value in nonunion management.

### Challenges and limitations of protein therapy

Beyond their side effects, the high cost of recombinant proteins and monoclonal antibody therapies remains a major barrier to widespread clinical adoption.^121^ The price of treatment varies depending on the specific protein, fracture type, dosage, and healthcare setting. For instance, the costs of the commercial BMP-7 product Osigraft® were between €15 000 and €18 000 per patient for tibial shaft nonunions before its production was discontinued.^122^

Therapeutic proteins are often produced in non-human systems (e.g., bacteria or yeast) that lack the necessary cellular machinery for human-specific post-translational modifications, such as glycosylation, phosphorylation, or acetylation—modifications essential for proper protein function.^123^ Another major challenge is maintaining effective protein levels at the fracture site, as their short half-life often necessitates repeated administrations.^124^ Recombinant proteins are highly susceptible to denaturation during production, storage, and delivery, leading to misfolding or partial unfolding, which significantly reduces their biological activity.^125^ These limitations further hinder their feasibility for routine clinical use, highlighting the need for advanced delivery strategies that enhance protein stability, bioavailability, and retention at the injury site.

### Gene therapy

Gene therapy aims to introduce genes encoding therapeutic proteins into target cells, enabling them to endogenously produce one or more desired proteins at the site of injury with native conformation and proper post-translational modifications.^23^ This approach has the potential to overcome the limitations of recombinant protein therapy by ensuring sustained, physiologically relevant levels of therapeutic proteins directly at the fracture site.^126^ Additionally, since a single copy of plasmid DNA (pDNA) can give rise to multiple copies of the therapeutic protein, gene therapy offers an efficient way to amplify protein production. While this enhanced endogenous protein production is certainly an advantage, a challenge of this approach compared to protein therapy, where precise dosing is possible and controlled protein delivery is feasible, is that the exact amount of protein copies that a DNA molecule produces is unknown, which could be a regulatory challenge when it comes to describing the immediate bioavailability of the active agent.

Gene delivery can be achieved using viral (transduction) or non-viral (transfection) vectors, through either an in vivo or ex vivo gene-transfer strategy.^126^ Both methods have demonstrated promising results in preclinical models.^127^ Among all growth factors, BMP genes have been extensively studied for bone healing, with gene therapy-based delivery showing encouraging outcomes in animal studies.^128–130^ Adenoviral vectors, in particular, have been used to achieve prolonged gene expression at the fracture site.^131^ However, gene therapy for bone regeneration has seen limited clinical application, with only four trials conducted to date, all in the Russian Federation.^132^ The first trial (NCT02293031), initiated in 2014, evaluated a gene-activated bone substitute incorporating a collagen–hydroxyapatite scaffold loaded with a VEGFA-encoding plasmid.^133^ Results showed improved bone density within three months and no reported adverse effects. However, the trial was withdrawn due to financial constraints.^134^

A subsequent trial (NCT03076138) by the same research team investigated an octacalcium phosphate (OCP) scaffold carrying a VEGFA plasmid for maxillofacial bone regeneration. The study, involving 20 patients, demonstrated successful bone formation with no complications.^132^ These promising outcomes led to regulatory approval in Russia in 2019, making this technology available for clinical use in oral and maxillofacial surgery. Another trial (NCT04705857) investigated a gene-activated matrix combining a demineralized bone allograft with a dual cassette vector expressing VEGFA and BMP-2 for treating ulnar pseudarthrosis.^135^ Results showed enhanced bone regeneration without adverse effects. The most recent ongoing trial (NCT04511689) is evaluating gene-activated bone substitutes containing OCP and VEGFA-expressing DNA against a xenogeneic deproteinized bone matrix for alveolar ridge augmentation before dental implantation. The trial is still in progress, with results yet to be published.

Despite its promise, gene therapy remains expensive and faces considerable regulatory hurdles.^126^ Treatments can cost an estimated $1 million or more, primarily due to the high expenses associated with viral vector production.^25^ Viral vectors are frequently used to deliver DNA because they more efficiently cross the nuclear membrane—especially in non-dividing cells—than non-viral alternatives. However, regulatory concerns persist regarding the use of viral vectors, particularly the risk of transgene integration into the host genome, which can lead to potentially dangerous insertional mutagenesis effects.^127^ Consequently, further research is needed to establish the long-term safety and efficacy of gene therapy for bone healing.

### Transcript therapy

While protein replacement therapy and gene therapy hold promise for bone regeneration, their clinical translation remains challenging due to financial, safety, and regulatory hurdles.^136^ This is reflected in the limited number of FDA-approved recombinant proteins for bone repair^137^ and the scarcity of approved gene therapy protocols targeting musculoskeletal disorders—currently, only two gene therapies are approved for osteoarthritis and disc degeneration.^138^ To address these challenges, a newer category of gene therapy, transcript therapy, has emerged. Instead of using pDNA, this approach delivers messenger RNA (mRNA) encoding the therapeutic protein. Transcript therapy offers several advantages, including reduced risk of genomic integration and potentially lower manufacturing costs, making it a promising alternative for safer and more affordable gene-based treatments for bone fractures.^139^

Transcript therapy relies on chemically modified mRNA (cmRNA) to enhance protein production. Structural modifications to mRNA, including adjustments to the 5′ cap, untranslated regions (UTRs), poly-A tail, and modified nucleotides, enhance stability, increase translation efficiency, and reduce immunogenicity.^140^ This approach enables prolonged protein synthesis (lasting 1–2 weeks) while ensuring proper native conformation and post-translational modifications—a key advantage over recombinant protein therapies.^141^ Additionally, cmRNA mitigates safety concerns associated with viral vectors in DNA-based gene therapy.

To improve cmRNA efficacy, researchers have focused on both sequence engineering and delivery strategies. One common approach to enhance translation is pyrimidine substitution, where uridine and cytidine are partially replaced with for instance 2-thiouridine and 5-methylcytidine.^47^ This modification reduces Toll-like receptor (TLR) and RIG-I recognition, minimizing immune activation and inflammation.^142^ Another optimization involves minimizing the 5′-UTR, as excessive secondary structures can hinder ribosome binding and slow translation. For example, Zhang et al. demonstrated that incorporating a translation initiator of short untranslated regions (TISU) into BMP-2 cmRNA significantly improved mRNA stability and transfection efficiency, leading to robust BMP-2 expression.^143^

Like all nucleic acid-based therapies, cmRNA delivery remains a major challenge due to its highly anionic nature and susceptibility to RNase degradation. To achieve efficient cellular uptake, cmRNA must be encapsulated in carriers with strong cell-penetrating properties. Among the various delivery systems, cationic lipids have shown the most promise as non-viral vectors for mRNA transport. Lipid nanoparticles (LNPs) have been successfully utilized to deliver BMP-2 cmRNA for bone regeneration.^144^

Beyond lipid-based vectors, researchers have explored cationic polymers—such as polyamines, block copolymers, and polypeptides—for mRNA delivery. However, few polymeric systems have been applied to bone regeneration. Interestingly, one study found that cmRNA transfection of human MSCs, fibroblasts, and osteoblasts using the commercial polymeric vector TransIT-X2 resulted in significantly lower protein expression compared to lipid-based vectors.^145^ In contrast, pDNA transfection with the same polymeric vector resulted in higher protein expression, highlighting the need for further optimization of polymeric carriers for efficient cmRNA delivery in bone repair applications. In this context, studies have demonstrated that bone tissue formation can be induced by delivering BMP-2/NS1 mRNAs loaded into lipopolyplexes. These are hybrid delivery alternatives, consisting of polycations that condense nucleic acids and lipid components that coat the polyplex to enhance membrane interaction and cellular uptake.^146^

Previous studies have leveraged cmRNA encoding BMP-2 in combination with biomaterials to create transcript-activated matrices (TAMs) for osteogenic differentiation. For instance, researchers have combined BMP-2 cmRNA with fibrin gel and micro-macro biphasic calcium phosphate (MBCP) granules to construct TAMs.^147^ These materials facilitated sustained release of hBMP-2 cmRNA for 7 days, with a faster release observed from MBCP granules compared to fibrin gels. To further demonstrate the potential of biomaterial-assisted cmRNA delivery for bone regeneration, another study explored cmRNA-based BMP-2 coatings on titanium implants, assessing different coating strategies and biomaterial incorporation techniques.^148^ Researchers applied cmRNA coatings using three biocompatible materials: poly(d,l-lactic acid) (PDLLA), fibrin, and fibrinogen. These coatings significantly improved transfection efficiency by delaying cmRNA release.

Several studies have demonstrated the effectiveness of cmRNA encoding BMP-2, BMP-7, and BMP-9 in promoting bone healing across in vitro and in vivo models.^47,149,150^ Among these, BMP-2 cmRNA has been the most widely explored. A recent study by De La Vega et al. examined the use of optimized BMP-2 cmRNA for healing large segmental osseous defects in rats.^150^ The cmRNA remained localized at the defect site, generating sustained BMP-2 expression for several days. Notably, bone defects healed with doses as low as 25 μg of BMP-2 cmRNA, and 50 μg treatments successfully bridged defects within 4 weeks—without the excessive callus formation observed with recombinant human BMP-2. This study highlighted cmRNA’s ability to accelerate fracture healing while promoting natural bone remodeling compared to recombinant proteins.

An innovative approach also explored the co-delivery of cmRNA encoding VEGF and Runx2. When applied to rat mandibular defects, this combination significantly enhanced vascularization and bone formation.^151^ This strategy highlights the potential of multi-gene cmRNA therapies and extends beyond traditional growth factor delivery by incorporating transcription factors, offering new alternatives that can further be explored to enhance bone formation.

Gene therapy has historically been viewed as a high-risk and costly intervention, subject to strict regulatory scrutiny, particularly in non-lethal conditions such as musculoskeletal injuries.^152^ The lengthy and expensive approval process has contributed to its limited adoption.^153^ However, cmRNA therapy has the potential to shift this perception by offering a safer and more affordable alternative, particularly in bone regeneration, where cost-effective solutions are crucial to improving the lives of millions affected by fractures.

Despite its promise, cmRNA therapy still faces significant challenges. Comprehensive pharmacokinetic, toxicological, and biodistribution studies will be essential before clinical translation. Additionally, demonstrating efficacy in large-animal models will likely be a prerequisite for advancing to human clinical trials.

### microRNA for fracture healing

MicroRNAs (miRNAs) are a class of small, non-coding RNA molecules, approximately 22 nucleotides in length, that play a pivotal role in post-transcriptional gene regulation.^154^ They influence a wide range of cellular processes—including differentiation, proliferation, and apoptosis^155^—and have emerged as critical regulators of osteogenesis by modulating the expression of genes involved in osteoblast differentiation and function.^156,157^ Numerous miRNAs have been identified as either promoters or inhibitors of bone formation, acting through various molecular targets and signaling pathways, for example (Table 2).

**Table 2:** Key MicroRNAs Involved in Bone Regeneration

miRNA	Target(s)	Mechanism of Action	Preclinical Findings
miR-26a	GSK-3β	Enhances Wnt/β-catenin signaling by repressing GSK-3β, promoting osteoblast differentiation.^279^	Overexpression significantly improves osteoblast differentiation and in vivo bone regeneration.^161^
miR-21	PTEN, Spry1, Smad7	Promotes osteogenesis via activation of the PTEN/PI3K/Akt/HIF-1 pathway; suppresses anti-osteogenic factors.^280^	Facilitates migration and osteogenic differentiation of MSCs and increases bone formation in critical-size defects.^280^
miR-378	p110α, MyoR, Fus-1, Vimentin, CASP3	Activates the sonic hedgehog (Shh) signaling pathway by negatively regulating its inhibitor, Sufu.^281^ Attenuates high glucose-suppressed osteogenic differentiation through targeting CASP3 and activating PI3K/Akt signaling pathway.^282^	Supports the coupling of osteogenesis and angiogenesis upregulating VEGF secretion.^283^
miR-495	HMGA2	Acts as a negative regulator of bone formation by targeting HMGA2 (High Mobility Group AT-hook 2).^284^	Its downregulation has been associated with improved femoral healing in murine models.^284^
miR-125b	STAT3, p53, RUNX2, ERBB2	Regulates both osteoblast and osteoclast activity. Targets and suppresses the expression of the transcriptional repressor PRDM1 in osteoclast precursors.^285^	Exhibits context-dependent effects: enhances mineralization under dexamethasone stimulation but inhibits it under calcium treatment.^286^ Improves age-related changes in bone mass and quality.^287^
miR-133a	RUNX2, CTGF	Inhibits osteoblast differentiation by directly targeting RUNX2, a master regulator of osteogenesis.^288^	Downregulation promotes osteogenic differentiation of MSCs and enhances bone formation.^288^
miR-148b	Wnt1, Smad2	Enhances Wnt signaling (targeting Wnt1) and inhibits TGF-β/Smad2 signaling.^289^	Overexpression enhances osteogenesis, improving bone formation and mineral density in in vitro and in vivo models.^290,291^

To deliver therapeutic miRNAs, both viral and non-viral vector systems have been explored. Viral vectors, such as lentiviruses, offer high transfection efficiency and stable gene expression, but carry risks including insertional mutagenesis and immunogenicity, which limit their clinical application.^158^ In contrast, non-viral vectors—such as nanoparticles, liposomes, and exosomes—provide a safer alternative with tunable properties for targeted and controlled miRNA delivery.^159,160^

Additionally, miRNA-functionalized scaffolds made from biomaterials like collagen, chitosan, or tricalcium phosphate have been engineered to promote localized and sustained osteogenic effects, showing promising results in preclinical models of bone regeneration.^160–163^

Despite these advances, challenges such as susceptibility to enzymatic degradation, off-target effects, and the development of efficient, tissue-specific delivery systems must be addressed before miRNA-based therapies can be translated into routine clinical practice.

### Peptides

Peptides are short amino acid oligomers derived from the active domains of proteins, which often lack a stable 3D structure. They can be derived from larger proteins or designed de novo. They present a cost-effective and easily synthesizable alternative to protein therapy.^164^ These peptides typically function by binding to high-affinity receptors on target cells, enhancing bioavailability and targeted delivery to fracture sites.

Several peptides have been engineered to enhance osteogenic responses, many of which are derived from growth factors and bone-related proteins. Lactoferrin-derived peptides and calcitonin gene-related peptides, for example, have been reported to promote fracture healing and enhance bone formation in vivo.^164,165^ Another interesting peptide is thrombin peptide 508 (TP508), or Chrysalin, which maintains thrombin’s regenerative properties without its clotting effect, enhancing osteoblast proliferation, differentiation, and chemotaxis.^166,167^

Peptides serve various functions in bone regeneration, such as promoting cell binding, enhancing osteoblast adhesion, and facilitating scaffold integration.^168^ P-15, a 15-amino acid sequence identical to the cell-binding region of collagen type I, improves cell attachment to bone substitutes and stimulates extracellular matrix production.^169^ RGD-containing peptides support osteoblast adhesion, proliferation, and osteogenic marker expression on scaffolds and graft materials.^170,171^ Meanwhile, self-assembling peptides, often described as “molecular Lego”, feature alternating hydrophilic and hydrophobic amino acids, enabling the formation of nanofiber matrices that enhance cell adhesion, osteoblast differentiation, and bone volume.^172,173^

For a comprehensive review of clinical and experimental studies on peptides in bone regeneration, the literature article by *Puntos* et al. provides an in-depth analysis of their therapeutic potential.^168^

### Small molecules

Small molecules with bone regenerative potential have gained attention due to their efficacy, stability, and cost-effectiveness.^174,175^ Their small size (<1 000 Da) reduces the likelihood of triggering immune responses,^176^ and unlike protein therapies that depend on maintaining a specific three-dimensional structure for bioactivity, small molecules are typically less susceptible to structural changes and retain their function under a wider range of conditions.^177,178^ Over the last years, advances in high-throughput screening have led to the discovery of numerous osteoinductive small molecules.^179–181^

Among the most studied is simvastatin, which has demonstrated increased body weight, bone mineral content, and mechanical strength in osteoporotic animal models.^182,183^ The osteogenic oxysterol Oxy133 has shown comparable efficacy to recombinant human BMP-2 in spinal fusion while avoiding its adverse effects.^184^ Interestingly, FTY720, a sphingosine-1-phosphate analog, has shown contradictory results in fracture healing studies. While some research suggests that local delivery of FTY720 enhances both bone formation and microvascular development in a rat cranial defect model,^185,186^ another study reported that systemic administration of FTY720 did not significantly improve bone regeneration in a murine fracture model.^187^ These findings highlight the importance of delivery strategy, as the therapeutic efficacy of small molecules like FTY720 may depend heavily on their mode and site of administration.

Another notable molecule, FK506 (tacrolimus), functions as an immunosuppressant by inhibiting calcineurin and blocking T-cell activation in transplant recipients.^188^ A study by De la Vega et al. investigated FK506 in rat femoral critical-size defects using both unmodified and adenovirally transduced MSCs expressing BMP-2.^189^ Interestingly, defects did not heal in the presence or absence of FK506 when unmodified cells were used. However, when combined with BMP-2-expressing cells, FK506 led to complete defect healing, resulting in a slimmer callus, improved cortication, and advanced marrow reconstitution. These findings suggest that FK506 alone may not significantly influence intrinsic bone healing but can enhance the regenerative response when paired with BMP-2-expressing MSCs.

Additionally, other small molecules have also demonstrated significant osteoinductive potential. For instance, the small-molecule BMP activator SVAK-12 markedly accelerated fracture healing in a rat femoral fracture model without requiring exogenous rhBMPs.^190^ Moreover, some small compounds have been evaluated in large animal models, further supporting their translational potential. One such example is calcium phosphate-coated implants releasing bisphosphonates, which enhanced periprosthetic bone density and implant integration in an osteoporotic sheep model, underscoring their promise in orthopedic applications.^191^ Beyond these, numerous small molecules have been investigated for their osteogenic properties. Zhang et al. reviewed various small-molecule amides, detailing their biochemical properties and regulatory mechanisms in bone regeneration.^192^

These findings underscore the potential of small molecules as scalable, stable, and cost-effective alternatives to protein-based therapies, offering innovative strategies for bone repair and regeneration.

### Cell therapy and platelet-based strategies

#### Cell therapy

Cell therapies involve transplanting stem cells, progenitor cells, or differentiated cells, which can come from processed host tissue, culture-expanded stem cells, purified populations of stem cells, or genetically modified stem cells.^193^ These therapies are appealing for treating impaired bone healing because they are typically autologous, meaning they come from the patient’s own body, thus minimizing the risk of rejection.^194^

Stem cells have the capacity to proliferate and differentiate into distinct cell lineages, including osteoprogenitors. The most abundant sources of stem cells are adipose tissue and bone marrow, however they can be found in other tissues in smaller numbers (peripheral blood, muscle, dermis, etc.).^195,196^ Several studies have demonstrated the efficiency and safety of MSCs in bone healing in clinical settings. In a pilot clinical study, Marcacci and colleagues treated four patients with diaphyseal bone defects using autologous bone marrow-derived osteoprogenitor. Complete healing was achieved in all patients (two with ulna defects, one with a humerus defect, and one with a tibial defect) within six months, with no reported complications over a 6–7-year follow-up period.^197^ Quarto et al. successfully treated large bone defects in three patients using autologous bone marrow-derived MSCs on a ceramic scaffold. Radiographs and computed tomography scans showed significant callus formation and good integration with the host bone by the second month after surgery.^198^ Additionally, in a multicenter clinical trial, 64 patients with delayed long bone fracture healing were treated with autologous cultured osteoblasts or no treatment. The osteoblast group showed a significant increase in radiographic callus formation at two months compared to controls, highlighting the potential of cellular therapies in bone repair.^199^

A common option to deliver stem cells is via bone marrow aspirates (BMAs). These aspirates contain a heterogeneous population of cells, including stem cells, progenitor cells, and hematopoietic cells. Thus, BMAs contain cells capable of differentiating into osteoblasts when exposed to osteoinductive signals and have been employed to treat delayed unions and nonunions.^200,201^ While the iliac crest has traditionally been the primary site for bone marrow aspiration, other sites such as the vertebral body, proximal humerus, proximal and distal tibia, calcaneus, and fibula have also been used.

Despite their promise, cell therapies face significant challenges. Isolating and expanding MSCs can be labor-intensive, particularly in older patients, where stem cell numbers and function decline.^202^ Ensuring consistent differentiation into osteogenic lineages remains a hurdle, as factors such as the donor’s age, health status, and genetic background can influence the behavior and regenerative potential of the cells.^203^ This variability can lead to inconsistent outcomes that influence cell fate. Moreover, the high costs of cell isolation, expansion, and regulatory compliance limit widespread clinical application. There is also a potential risk of unwanted differentiation or tumorigenesis, especially with induced pluripotent stem cells (iPSCs).^204^ Nonetheless, advances in cell engineering, biomaterials, and bioreactors continue to improve the feasibility of cell-based approaches for bone regeneration.

Interestingly, most current clinical trials for nonunions, registered in ClinicalTrials.gov, are exploring cell-based strategies. Cellular mechanisms with their multifaceted regenerative capabilities— including secreting growth factors, modulating inflammation, and directly contributing to tissue formation—offer a comprehensive approach to tackling the biological complexities of nonunion. Table 3 provides a detailed overview of various clinical trials currently registered for bone regeneration in nonunions. While challenges related to standardization, scalability, and long-term safety persist, ongoing research and the increasing number of clinical investigations highlight cell therapy as a cornerstone of future nonunion treatment strategies.

**Table 3:** Clinical trials using biologics to treat nonunions

Biologic	Details	Place	ID /Status
Human Amniotic Epithelial Cells (hAECs)	- For limbs nonunions. - hAECs obtained from the epiblast as early as 8 days after fertilization. - 50 million cells transplanted to nonunion site after debridement surgery.	Shanghai, China	NCT03031509 Unknown
Umbilical Cord Blood Mononuclear Cells (UCB-MNCs)	- For traumatic noninfectious bone nonunions or delayed bone unions, with no obvious callus growth 8 months after local bone grafting. - 2 mL with 100 million cells, once every two weeks, 3 times in total.	Jinan, Shandong, China	NCT04997590 Unknown
Mesenchymal Stem Cells (MSCs)	- For tibial and femoral nonunions having no radiological callus after 6 months. -2-3 mL with approximately 40 million cells.	Mashhad, Khorasan, Iran,	NCT01788059 Completed
Bone Marrow-derived Mesenchymal Stem Cell (BMSCs)	- Nonunion or delayed union with more than 4 cm distance to joint.	Tehran, Iran	NCT01206179 Completed
Reamer Irrigator Aspirator (RIA) vs Autogenous Iliac Crest Bone Graft (AICBG)	- For nonunion of a long bone requiring bone grafting. - RIA to harvest bone graft from the femoral canal.	Toronto, Canada	NCT01382485 Unknown
Adipose Tissue Derived Stromal Vascular Fraction (SVF)	- For nonunion of long bones or delayed union diagnosed with more than 4 cm distance from the joint. - Heterogeneous cell population (20% CD105^+^ MSCs, 40% CD34^+^ hematopoietic cells).	Rudrapur, India	NCT04340284 Completed
Bone Marrow Mononuclear Cells	- For long bone nonunion. - Obtained by posterosuperior iliac crest aspiration and isolated by Ficoll density gradient.	Oviedo, Spain	NCT01581892 Completed
Bone Marrow Mononuclear Cells	- For long bone nonunion of lower extremities (femur and tibia). - Implanted in collagenic 3-D scaffold with BMP-2.	Khorasan, Iran	NCT01958502 Unknown
NVD-003 (Autologous stem cells)	- For lower limb nonunion. - Stem cells embedded in their self-secreted extracellular matrix, combined with hydroxyapatite/beta-tricalcium phosphate particles, cocktail of growth factors, and miRNAs.	Belgium, Luxembourg, Switzerland	NCT06335394 Active
XCEL-MT-OSTEO-ALPHA	- ex vivo expanded autologous MSCs For atrophic nonunion of long bones. - Fixed in allogenic bone tissue.	Barcelona, Spain	NCT02230514 Completed
Tissue-Engineered Bone (with BMSCs)	- For bone nonunion caused by tumor or trauma. - BMSC in porous β-tricalcium phosphate scaffold. - 3.4 million cells in 10 mL serum-free medium seeded onto scaffold and co-cultured for two weeks.	Xi’an, China	NCT02748343 Unknown
BMSCs	- For post-traumatic nonunion. - Cells cultured are seeded on a collagen scaffold, included into autologous platelet-rich plasma (PRP) clot.	Caracas, Venezuela	NCT06103396 Active
Preosteoblast Cells	- For long bone nonunion. - Cells obtained from 50 mL of bone marrow harvest and culture for 3 weeks.	Liege, Belgium	NCT00916981 Completed
MSCs	- For atrophic nonunion of long bones. - Patient transplanted with stem cells and hydroxyapatite.	Jakarta, Indonesia	NCT01626625 Unknown
Vivigen Cellular Bone Matrix	- Contains lineage-committed bone cells (osteoblasts and osteocytes) within a corticocancellous bone matrix and demineralized bone. - For acute fracture, delayed, nonunion.	Several locations in the US	NCT04299022 Active
Autologous BMSC Transplantation in Combination with Platelet Lysate	- For nonunion fracture of tibial midshaft.	Tehran, Iran	NCT02448849 Unknown
2 Doses of Autologous BMSC+Biomaterial vs Iliac Crest AutoGraft	- For humerus, tibial or femur diaphyseal or metaphysodiaphyseal fracture nonunions. - Low Dose BMSC: 100 million cells. - High Dose BMSC: 200 million cells. - Biomaterial: biphasic calcium phosphate granules.	Multiple locations in Germany, Italy, and Spain	NCT03325504 Completed
Mononucleotide Autologous Stem Cells	- Tibial or femur nonunions. - A demineralized bone matrix using Ignite ®ICS injectable scaffold manufactured by Wright Medical Technology.	Jerusalem, Israel	NCT01435434 Unknown
rhBMP-2 or rhBMP-7	- For acute long bone fractures or nonunions.	Munich Germany	NCT05065684 Completed
rhBMP-2 vs Autologous Bone Grafting	- For nonunion of the docking site of long bones.	Leuven, Belgium	NCT01756144 Unknown
Gene-activated Bone Substitute	- For long bone nonunions: Gustilo I and II, IIIA and IIIB humerus, tibial or femur fracture. - Gene-activated matrix based on octacalcium phosphate and plasmid DNA encoding VEGFA gene, mixed with shredded autobone.	Saint Petersburg, Russian Federation	NCT04705857 Unknown

#### Platelet-based strategies

A relatively novel approach in bone regeneration involves platelet-rich plasma (PRP), a blood-based therapy utilizing platelets rich in growth factors and cytokines that play a crucial role in the early phases of bone regeneration.^205^ However, PRP research is still in its early stages, and its efficacy as a standalone treatment remains unclear, as it is often used in combination with other adjuvants.

PRP is typically obtained by drawing patient’s blood into a tube containing an anticoagulant, followed by centrifugation. In some cases, it is further treated with calcium chloride and bovine thrombin to induce platelet activation and form a gel-like matrix for direct application. This activation triggers platelet degranulation, leading to the sustained release of growth factors, while the gel serves as a biological carrier that prolongs their availability at the treatment site.^206^ Since PRP is autologous, it is generally safe and non-immunogenic. However, the use of bovine thrombin carries a risk of autoantibody formation and coagulopathies.^207^

In vitro studies have shown that PRP promotes MSCs proliferation and osteogenic differentiation, though excessive concentrations may have an inhibitory effect.^208^ Clinical trials have produced mixed results. For instance, one study comparing PRP-enhanced bone grafts to standard bone grafts in spinal fusion found no difference in nonunion rates, leading the authors to advise against PRP use.^209^ Conversely, another study on tibial osteotomies reported faster union in the PRP-treated group, yet no long-term functional benefits were observed.^210^

Currently, PRP remains an exploratory therapy for bone healing. Due to inconsistent clinical outcomes and uncertainties regarding optimal concentration, it cannot yet be recommended for routine fracture treatment.

### Synergistic potential with advanced biomaterials

The therapeutic efficacy of biologics for bone regeneration can be significantly amplified by their combination with biomaterials. Beyond simply serving as inert delivery vehicles, biomaterial scaffolds are designed to provide crucial structural support for defect filling, offering a temporary template for new bone formation.^211^ More importantly, these materials can be engineered to precisely control the spatial and temporal release kinetics of incorporated biologics, ensuring sustained and localized concentrations at the repair site.^212,213^ This controlled delivery minimizes systemic exposure, reduces off-target effects, and optimizes the therapeutic window for cellular responses.

Furthermore, biomaterials can exert intrinsic biological cues that actively participate in the regenerative process.^214^ In particular, scaffolds composed of bioactive ceramics, polymers, or composites can provide critical structural support in bone defects, mimicking the native extracellular matrix and creating a permissive environment for cell infiltration, attachment, and differentiation.^215–217^ Their surface properties, pore architecture, and mechanical characteristics can be tailored to influence cell adhesion, proliferation, differentiation, and migration, thus guiding the host’s endogenous regenerative machinery.^147,218^ When integrated with biologics, such as growth factors or cells, these interactive scaffolds create a microenvironment that not only presents the therapeutic agent but also provides the necessary physical and chemical signals to profoundly enhance osteoinduction, osteoconduction, and overall tissue integration, thereby maximizing the potential for successful bone regeneration.^219,220^

## Potential clinical applications

**Fig. 4:**
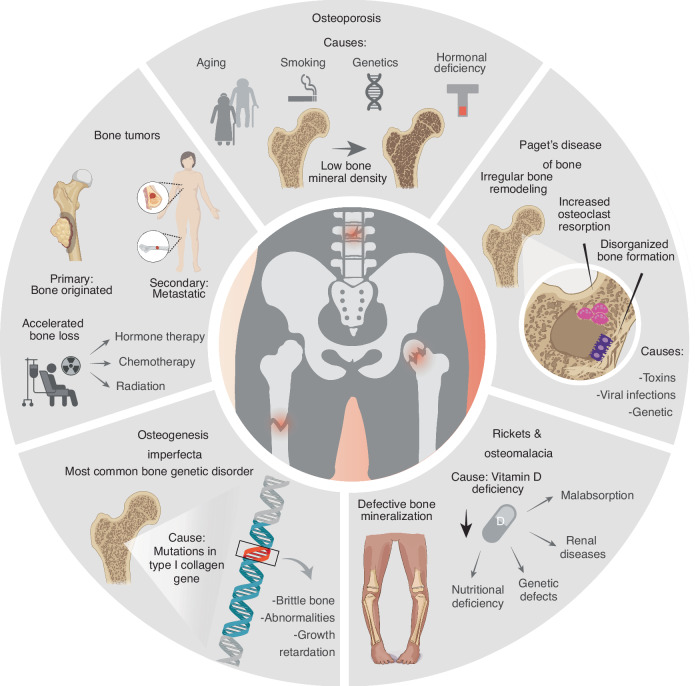
Pathological conditions associated with difficult-to-heal bone fractures. Several bone diseases, including osteoporosis, Paget’s disease of bone, rickets, osteomalacia, osteogenesis imperfecta, and bone tumors, predispose patients to recurrent fractures with delayed or failed healing. This schematic highlights the key characteristics of each condition. Patients affected by these disorders present deficiencies in bone metabolism, structural integrity, or cellular function; therefore, they may particularly benefit from biologic therapies designed to stimulate bone regeneration

The use of biologics is not limited to patients suffering from severe traumatic injuries that result in nonunion fractures. Biologic therapies are also highly relevant for individuals with degenerative and genetic bone diseases, which can predispose them to non-self-healing fractures (Fig. 4). In the following sections, we discuss key clinical applications where biologics have already shown, or are expected to have, a significant impact in enhancing fracture healing.

### Osteoporosis

Osteoporosis is characterized by low bone mineral density and deteriorated bone microarchitecture, leading to increased bone fragility, particularly among the elderly and postmenopausal women.^221^ Individuals with osteoporosis face a high risk of fractures, which can be physically debilitating and contribute to a decline in overall health and quality of life.^222^ The most common form, primary osteoporosis, occurs naturally with aging, whereas secondary osteoporosis results from underlying diseases or long-term medication use.^223^

Osteoporotic fractures, particularly those affecting the spine, pelvis, or hip, are associated with increased morbidity and mortality.^224^ Most epidemiological studies have focused on hip fractures, which have the most severe sequelae and are the easiest to study since most patients undergo hospitalization. Contrarily, vertebral fractures are harder to track as they can be asymptomatic.^224^

Biologic therapies such as denosumab, a monoclonal antibody that inhibits osteoclast activity, have been shown to reduce fracture risk in postmenopausal osteoporosis.^225^ Other anti-resorptive therapies include bisphosphonates, which increase bone mineral density and reduce fracture incidence.^223^ Additionally, biologics like romosozumab (Evenity®), which target sclerostin, exhibit both anabolic and anti-resorptive effects. This dual mechanism makes romosozumab particularly effective for treating osteoporosis by simultaneously promoting bone formation and reducing bone resorption.^226–228^

Emerging gene therapy strategies for osteoporosis have shown promise in preclinical studies, where engineered cells overexpressing BMP-2 or type I collagen successfully increased bone mass and accelerated fracture healing in osteoporotic animal models, including rats and sheep.^229–231^ Beyond conventional DNA-based gene therapy, cmRNA approaches are also being explored for their therapeutic potential in osteoporosis. For instance, a study by Liu et al. demonstrated that systemic delivery of 7-methylguanosine (m⁷G)-modified Runx2 mRNA using bone-targeted lipid nanoparticles effectively enhanced bone formation in senile osteoporotic mice, highlighting cmRNA as a promising and translatable strategy for treating osteoporotic-related fractures.^232^

### Rickets and osteomalacia

Two rather uncommon diseases prone to end up in fracture are rickets and osteomalacia, affecting children and adults, respectively. They are caused by defective bone mineralization due to vitamin D deficiency or genetic mutations affecting calcium and phosphate metabolism.^233^ Rickets results in skeletal deformities, such as bowed legs, due to impaired calcium and phosphate deposition during bone growth.^234^ In contrast, osteomalacia, which occurs in adults after bone growth has ceased, does not cause deformities but increases fracture risk, particularly in weight-bearing bones such as the pelvis, hip, femur and tibia.^235,236^

These conditions may arise from environmental factors, such as inadequate sun exposure, or genetic mutations affecting vitamin D metabolism.^237^ Vitamin D is synthesized in the skin upon sunlight exposure, so individuals living in high latitudes or those with chronic illnesses that limit outdoor activity are at increased risk.^233^ Additionally, gastrointestinal diseases like gastrectomy and malabsorption syndromes can impair vitamin D absorption, further contributing to these disorders.^224^

X-linked hypophosphatemic rickets (XLH) is the most common inherited form of rickets, caused by mutations in the PHEX gene that lead to excessive production of fibroblast growth factor 23 (FGF23).^238^ This hormone reduces phosphate reabsorption in the kidneys, resulting in chronic hypophosphatemia and impaired bone mineralization. Treatment with burosumab (Crysvita), a fully human monoclonal antibody that inhibits FGF23, has been shown to increase serum phosphate levels and enhance skeletal mineralization.^239^ As such, burosumab represents a promising biologic therapy to support fracture healing in patients with XLH. Similarly, children with hypophosphatasia (HPP)—a rare genetic disorder caused by deficient tissue-nonspecific alkaline phosphatase (TNSALP)—may benefit from enzyme replacement therapy with asfotase alfa, a recombinant form of ALP.^240^ By hydrolyzing inorganic pyrophosphate (PPi), a natural inhibitor of mineralization, asfotase alfa restores proper bone mineral deposition, promoting improved healing of fractures and pseudofractures in patients with HPP.^241^

Overall, studies specifically targeting fracture healing in the context of rickets and osteomalacia remain largely unexplored, but cmRNAs offer a promising approach. These can be engineered to transiently express osteogenic factors such as Runx2, Osterix, or BMPs to promote bone formation, while simultaneously addressing the underlying metabolic deficiencies. For example, in osteomalacia, cmRNAs encoding functional 1α-hydroxylase (CYP27B1) could enhance the local conversion of inactive to active vitamin D (1,25(OH)₂D₃), boosting calcium absorption and osteoblast activity.^242^ In hereditary hypophosphatemic rickets, cmRNAs could be designed to encode FGF23 antagonists or regulators of phosphate reabsorption in the kidney, restoring phosphate balance and improving bone mineralization.^243^

### Paget’s disease of bone

Paget’s disease of bone is a progressive disorder of bone remodeling, characterized by excessive osteoclast activity, leading to rapid and disorganized bone formation.^244^ It commonly affects the spine, pelvis, legs, and skull, increasing susceptibility to bone pain, deformity, and fractures.^224^ The prevalence of Paget’s disease is approximately 1.3% among individuals aged 45–74, but many cases remain undiagnosed due to the asymptomatic nature of early-stage disease.^244^ Although the exact cause remains unclear, possible contributors include slow viral infections, environmental toxins such as arsenic and lead, and genetic predisposition, as familial aggregation is observed in 40% of cases.^245,246^

The incidence of Paget’s disease has declined in recent years due to the widespread use of effective anti-resorptive therapies such as bisphosphonates and calcitonin. Nonetheless, severe cases and untreated individuals remain at high risk of pathological fractures, particularly transverse femoral fractures, which are prone to delayed healing or nonunion.^247^ These clinical challenges arise from the hallmark features of Paget’s disease: aberrant bone remodeling characterized by excessive osteoclast-mediated resorption followed by disorganized and mechanically compromised bone formation. Interestingly, Dkk1 RNA and protein levels have been reported to increase in pagetic osteoblast and stromal cells,^248^ as well as in the circulating serum of affected patients,^248^ suggesting that Dkk1 may be a viable therapeutic target for modulating dysregulated bone formation.

While cell-based therapies remain underexplored in the context of Paget’s disease–related fractures, they present a compelling theoretical approach due to their capacity to both supply functional osteoprogenitors and modulate the local bone remodeling environment. For instance, delivering exogenous MSCs could augment the pool of functional osteoprogenitors at the fracture site, which might be critical given the dysregulated endogenous bone cell behavior in Paget’s disease.

Novel approaches using microRNA therapeutics offer promising avenues to enhance local bone regeneration and correct remodeling defects. For instance, inhibiting miRNAs like miR-223, which promotes osteoclast differentiation, may help suppress excessive bone resorption.^249^ Delivering these therapeutics locally via nanoparticles or scaffold-based systems could enable targeted, site-specific action while minimizing systemic side effects. Importantly, miRNA therapies are non-integrative, reversible, and can be tailored or combined with other biologics, making them a versatile and personalized strategy for treating fractures associated with Paget’s disease.

### Osteogenesis imperfecta

Osteogenesis imperfecta (OI) is one of the most common genetic bone disorders, characterized by mutations affecting type I collagen production, resulting in bones that fracture easily.^250^ The severity of OI varies across subtypes; while mild cases may be asymptomatic, more severe forms are associated with frequent fractures, short stature or growth retardation, hearing loss, dental abnormalities, and ligament laxity.^251^ The most harsh cases generally lead to early death as the mutations are incompatible with life.^224^

Approximately 75% of OI-related fractures occur in childhood, but fractures in adulthood present additional challenges due to bone deformities and prior surgeries.^251^ Delayed unions and nonunion are often encountered and in such cases, implanting a bone graft is required with complex surgeries and frequent post-operative problems.^252^ Given the genetic basis of OI, personalized treatment strategies are essential. Some OI patients exhibit high bone mineralization, making their bones more brittle, rather than simply lacking bone mass.^253^ Gene and stem cell therapies are under investigation, with efforts focused on delivering wild-type collagen genes while silencing mutant collagen alleles using plasmid DNA and antisense gene therapy.^254,255^

Like its application in osteoporosis, therapies targeting sclerostin inhibition have shown promise in enhancing osteoblastic bone formation in OI. In a murine model of type III OI, treatment with an anti-sclerostin antibody significantly improved BMD, tibial cortical thickness, ultimate load, and stiffness.^256^ Furthermore, an open-label phase 2 clinical trial evaluated the pharmacodynamics and safety of BPS804, an anti-sclerostin antibody, in adults with moderate OI. Patients received three escalating doses (5, 10, and 20 mg/kg) at two-week intervals, while a reference group received no treatment. The therapy was well tolerated and resulted in increased bone formation, reduced bone resorption, and improved lumbar spine BMD.^257^

### Bone tumors and other malignancies

Bone tumors are abnormal growths within bone tissue that can be classified as either benign (non-cancerous) or malignant (cancerous). Malignant bone tumors are further categorized into primary tumors, which originate in the bone, and secondary tumors, which result from metastasis of cancers such as breast, prostate, and pancreatic cancer.^224^ Multiple myeloma is a common example of a secondary bone tumor, leading to bone destruction in over 80% of affected patients.^258^ Metastatic bone disease often affects the spine, pelvis, hip, femur, and skull, leading to severe pain, pathological fractures, and hypercalcemia.^259,260^

Fractures associated with bone tumors, whether primary or metastatic, pose significant clinical challenges due to persistent tumor-induced osteolysis and a compromised bone healing environment.^260^ In addition, cancer therapies—including hormone treatments, chemotherapy (particularly glucocorticoid-based regimens), and radiation—further reduce bone mineral density and accelerate bone loss. Addressing these complications requires a multidisciplinary strategy that not only promotes bone regeneration but also targets the underlying malignancy.^261^

mRNA therapeutics offer a particularly promising avenue. Beyond delivering cmRNAs encoding osteoinductive factors, cmRNAs can be engineered to express osteoprotegerin (OPG) or RANKL inhibitors, thereby limiting osteoclast-mediated bone resorption and restoring remodeling balance. Moreover, multi-targeted platforms could be designed to co-deliver anti-tumor mRNAs—such as those encoding immunostimulatory cytokines or apoptosis-inducing proteins—together with bone-regenerative cues, enabling localized and synergistic treatment of both the tumor and the fracture.^262^

Complementary to this molecular approach, bioactive scaffolds have shown strong clinical potential. For example, a study by Wang et al. introduced an innovative approach for reconstructing bone defects following the surgical excision of benign tumors in the lower extremities.^263^ The authors employed bioactive scaffolds combined with a proprietary stem cell rapid screening–enrichment–composite technology. When applied in clinical settings, these scaffolds seeded with enriched BMSCs significantly enhanced bone integration and healing, offering a promising and translational solution for improving postoperative outcomes in patients with tumor-related bone defects.^263^

## Conclusions

Biologic therapies represent a promising frontier in the treatment of nonunions. While protein and gene therapies have been explored for years, their clinical translation has been slow and limited. Emerging strategies, such as transcript therapy using chemically modified mRNA, along with advances in microRNA-based regulation, bioactive peptides, and small molecule modulators, offer a new wave of precise, controllable, and safer regenerative interventions.

As our understanding of bone regeneration at the molecular and cellular mechanisms deepens, we can develop more precise, biology-driven regenerative therapies. Future strategies should not only target osteogenesis but also incorporate key aspects of bone healing, including innervation, angiogenesis, and controlled inflammation, to promote the formation of fully functional bone tissue.

Personalized medicine is essential for optimizing biologic treatments, considering factors like age, sex, fracture type, and underlying bone pathologies. Advanced strategies could involve delivering a cocktail of transcription factors, growth factors, or ECM proteins delivered via transcript therapy with spatial and temporal gradients, along with miRNA mimics or inhibitors, mimicking natural bone healing, enabling precise control while maintaining low therapeutic doses. Additionally, hybrid approaches combining stem cell therapies may offer more effective and accelerated healing solutions.

To ensure widespread accessibility, cost-effective production methods and advanced delivery systems must be developed. Finally, robust clinical trials are necessary to validate the long-term safety and efficacy of these therapies across diverse patient populations and fracture types.
